# Hardness-and-Type Recognition of Different Objects Based on a Novel Porous Graphene Flexible Tactile Sensor Array

**DOI:** 10.3390/mi14010217

**Published:** 2023-01-14

**Authors:** Yang Song, Shanna Lv, Feilu Wang, Mingkun Li

**Affiliations:** 1 School of Electronic and Information Engineering, Anhui Jianzhu University, Hefei 230601, China; 2 Key Laboratory of Building Information Acquisition and Measurement Control Technology, Anhui Jianzhu University, Hefei 230601, China

**Keywords:** recognition, time sequence feature, hardness and type, porous graphene, flexible tactile sensor, residual network

## Abstract

Accurately recognizing the hardness and type of different objects by tactile sensors is of great significance in human–machine interaction. In this paper, a novel porous graphene flexible tactile sensor array with great performance is designed and fabricated, and it is mounted on a two-finger mechanical actuator. This is used to detect various tactile sequence features from different objects by slightly squeezing them by 2 mm. A Residual Network (ResNet) model, with excellent adaptivity and feature extraction ability, is constructed to realize the recognition of 4 hardness categories and 12 object types, based on the tactile time sequence signals collected by the novel sensor array; the average accuracies of hardness and type recognition are 100% and 99.7%, respectively. To further verify the classification ability of the ResNet model for the tactile feature information detected by the sensor array, the Multilayer Perceptron (MLP), LeNet, Multi-Channel Deep Convolutional Neural Network (MCDCNN), and ENCODER models are built based on the same dataset used for the ResNet model. The average recognition accuracies of the 4hardness categories, based on those four models, are 93.6%, 98.3%, 93.3%, and 98.1%. Meanwhile, the average recognition accuracies of the 12 object types, based on the four models, are 94.7%, 98.9%, 85.0%, and 96.4%. All of the results demonstrate that the novel porous graphene tactile sensor array has excellent perceptual performance and the ResNet model can very effectively and precisely complete the hardness and type recognition of objects for the flexible tactile sensor array.

## 1. Introduction

With the rapid development of microsensors, flexible electronic technology, artificial intelligence and sensor technology, as well as the urgent demand for intelligent robots in intelligent medical care, intelligent services and other fields, it is of great value and significance to carry out the research of intelligent robots [1,2,3,4]. Recently, researchers have been eager to assign robots the ability of tactile perception, similar to human skin, so as to improve the intelligence of robots. Flexible tactile sensors have wide application prospects in intelligent robot skin, wearable devices, health care and human–machine interaction fields [5,6]. Flexible tactile sensors can be used as robot skin to help intelligent robots perceive the physical properties of objects in real time, such as object hardness [7,8,9], roughness [10,11,12], temperature [13,14,15], and so on, which can effectively improve the robot’s perception of the surrounding environment and distinguish different objects; this is in order to make quick judgments and correct decisions in human–machine interactions, and improve the efficiency of practical applications [16,17,18].

In the previous research on object hardness recognition, the deformation characteristics of objects are usually explored by touching and pressing objects, collecting and processing the force, indentation displacement, tactile signals, tactile images and other data during the process, so as to achieve the recognition of object hardness attributes. For example, Liu et al. [19] proposed a variable motion mapping method to identify the hardness types of objects by calculating the ratio of force to indentation displacement. Kaim et al. [20] constructed a regression model to fit the trace of force-displacement observations, and used the slope of the regression line as a feature to classify the hardness of different objects. Yuan et al. [21] utilized the GelSight sensor to estimate the object hardness by comparing the changes in contact area and contact surface geometry, as well as the measured values of normal force during the press. Although the above methods could be easily implemented, the manually defined feature might lose some important feature information and, therefore, bias the classification result. Many researchers choose to use machine learning for the research of object hardness recognition. For example, Drimus et al. [22] presented a novel tactile sensor array based on flexible piezoresistive rubber, which was used to extrude the object and obtain the corresponding time series information. Then, the K nearest neighbor classifier, the objects were classified. Pastor et al. [23] used a novel high-resolution tactile sensor array to obtain pressure images at different grasping forces. These pressure images are used to feed a 3D Convolutional Neural Network (3D CNN), which is able to classify the grasped object. Hui et al. [24] designed a fusion model, based on a convolutional neural network (CNN) and a long short-term memory (LSTM) neural network, to extract the features of the tactile matrix sequence, and finally to recognize different objects.

In general, the hardness-and-type identification of objects mainly focuses on physical calculation and traditional machine learning methods. In this process, using the method of manually defining features may result in losing important feature information, which may lead to a deviation in the results. However, many studies [25,26,27,28] have shown that deep learning algorithms play an important part in the tactile recognition of intelligent robots. The tactile recognition method, based on deep learning algorithms, can extract useful features from input vectors and greatly reduce the computational complexity [29,30]. Based on our previous work [31], five deep learning algorithm models, the Residual Network (ResNet), Multilayer Perceptron (MLP), LeNet, Multi-Channel Deep Convolutional Neural Network (MCDCNN), and ENCODER, are constructed to conduct the recognition of the 4 hardness categories and the 12 object types; these are based on tactile sequence features, collected by the novel porous graphene flexible tactile sensor array, respectively. The average recognition accuracy of the 4 hardness categories, based on the five models, is 100%, 93.6%, 98.3%, 93.3%, and 98.1%. Meanwhile, the average recognition accuracies of the 12 object types, based on the same five models, are 99.7%, 94.7%, 98.9%, 85.0%, and 96.4%. The recognition accuracy of the ResNet model has been compared with those of the other four models. All the results demonstrate that the novel flexible tactile sensor array, proposed in this paper, is able to accurately perceive the tactile time sequence feature; in addition, the ResNet model with an excellent feature extraction ability and fairly good adaptivity can be very well applied to hardness-and-type recognition of different objects, based on the porous graphene flexible tactile sensor array.

## 2. Structure Design and Fabrication Procedure

**Figure 1:**
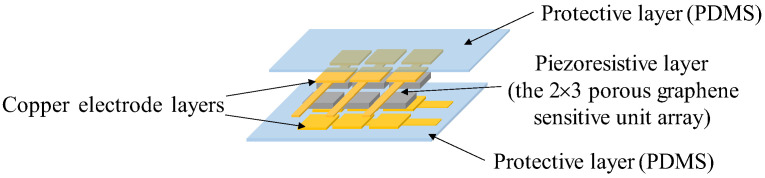
Structure of the porous graphene tactile sensor array.

Based on our previous work [31], the tactile signals from different objects could be collected by the novel porous graphene flexible tactile sensor array, fabricated in this paper. The structure of the sensor array, proposed in Figure 1, is mainly composed of three parts: the two protective layers, a piezoresistive layer, and the two copper electrode layers. Each protective layer is mainly made of a polydimethylsiloxane (PDMS) film, which can effectively prevent the sensitive unit array from being worn due to its excellent characteristics that include temperature resistance, high flexibility, good biocompatibility, and air permeability [32]; the piezoresistive layer is the core component of the sensor, which consists of a 2 × 3 porous graphene-sensitive unit array, and the solution impregnation method is used to fabricate the porous graphene-sensitive unit by depositing graphene oxide material on the porous PDMS frame; the two copper electrode layers include an upper electrode layer and a lower electrode layer, which are made of copper foil. Graphene material has excellent properties, such as high transmittance, a large specific surface area, high electron mobility, high elastic stiffness, and extensibility [33]. It has become a popular active material for the fabrication of flexible piezoresistive tactile sensors, and can greatly improve the sensitivity of tactile sensors [34]. Therefore, this paper utilizes graphene to prepare the porous graphene-sensitive units of the flexible tactile sensor array to ensure that the tactile information collected would be accurate and effective; this is in order to facilitate the implementation of hardness-and-type recognition experiments for different objects.

**Figure 2:**
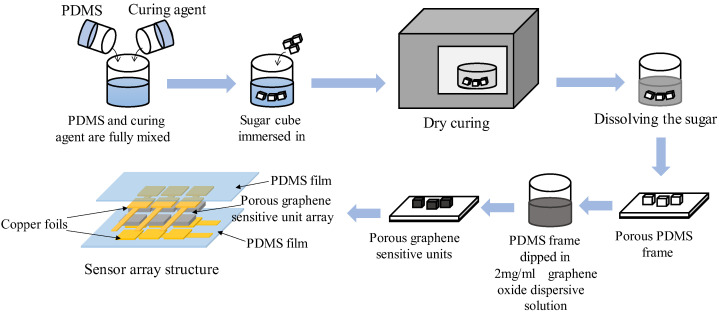
Fabrication procedure of the porous graphene flexible tactile sensor array.

**Figure 3:**
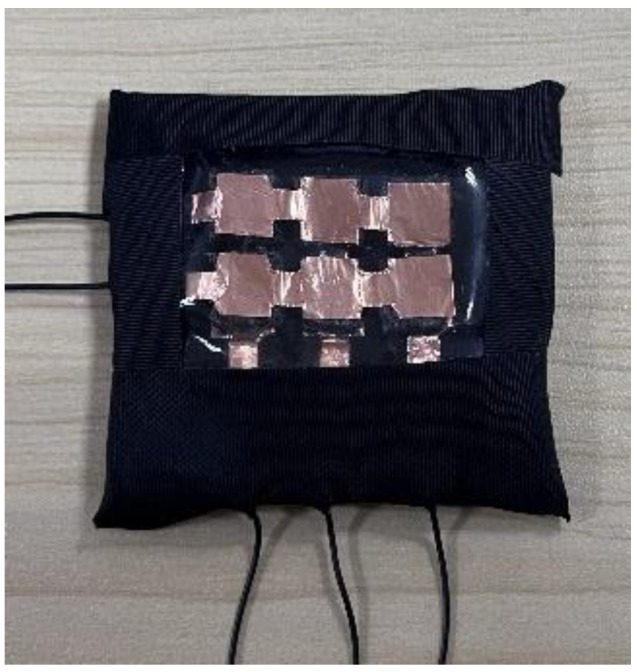
Prototype of the porous graphene flexible tactile sensor array.

The main ingredient of the sensor array comprises the 6 porous graphene flexible sensitive units; the size of each sensitive unit is 7 mm × 7 mm × 3 mm, and the horizontal distance between the two adjacent sensitive units is 3 mm. The sugar cubes, PDMS, curing agent, graphene oxide dispersive solution and copper foil are used to fabricate the porous graphene flexible tactile sensor array. The sugar cube is produced by Guangzhou Huatang Food Co. LTD; the PDMS (Sylgrad 184 silicone rubber) and the curing agents are two-component kit products, manufactured by Dow Corning Co.; the graphene oxide dispersive solution is produced by the Carbonene Technology Co. LTD, and its concentration is 2 mg/mL; the copper foil is produced by Wansheng Co. LTD, and its thickness is 0.06 mm. The specific fabrication procedure of the novel porous graphene flexible tactile sensor array is shown in Figure 2. Firstly, the PDMS and curing agent are fully mixed at a 10:1 ratio; secondly, a sugar cube is immersed in the mixed PDMS solution; thirdly, the sugar cube containing PDMS is put into a drying oven 3 h at 80 °C for curing; fourthly, the solidified PDMS sugar cube is immersed in hot water at about 60 °C to dissolve the sugar cube, and the porous PDMS frame is obtained; after that, the PDMS frame is immersed in a 2 mg/mL graphene oxide dispersive solution for 1 h, and then put it into a drying oven for 1.5 h at 55 °C for drying (this step is repeated 5 times to enable the graphene material to be fully deposited on the PDMS frame). Then, a flexible porous graphene-sensitive unit, with a size of 7 mm × 7 mm × 3 mm, is obtained. This fabrication process is repeated six times to produce six porous graphene-sensitive units to build the flexible tactile sensor array. Two copper foils are adhered to the upper and lower surfaces of the six porous graphene-sensitive units by conductive silver glue separately, which are used as electrode layers. Finally, PDMS films are used to encapsulate the copper foil electrode layers and the porous graphene-sensitive units as an entirety, then the flexible tactile sensor array is prepared. The prototype of the flexible tactile sensor array is shown in Figure 3.

**Figure 4:**
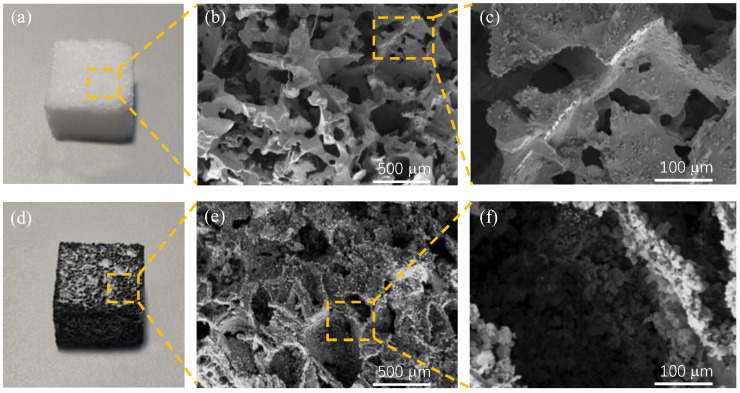
SEM micrographs of the porous graphene-sensitive unit and the porous PDMS frame (**a**) The porous PDMS sponge frame; (**b**) SEM micrograph of the porous PDMS sponge frame; (**c**) Magnified SEM micrograph of the porous PDMS frame; (**d**) The porous graphene-sensitive unit; (**e**) SEM micrograph of the porous graphene-sensitive unit; (**f**) Magnified SEM micrograph of the porous graphene-sensitive unit.

In this paper, scanning electron microscope (SEM) micrographs are used to analyze and study the surface morphology of the porous PDMS frame and the porous graphene-sensitive unit (shown in Figure 4). Figure 4a–c shows the physical picture of the porous PDMS frame and its SEM micrographs. As shown in Figure 4a–c, the prepared porous PDMS frame is a three-dimensional interconnected structure, and a large number of irregular pores of different sizes are formed inside the frame; the pore range is approximately 300 μm–600 μm. Figure 4d–f shows the physical picture of the porous graphene-sensitive unit and its SEM micrographs. As shown in Figure 4d–f, a large number of conductive graphene sheet materials are uniformly coated on the surface of the porous PDMS frame to form a three-dimensional conductive network. Finally, a flexible, foldable and stretchable conductor “porous graphene-sensitive unit” is obtained. When the porous graphene-sensitive unit is compressed under pressure, the graphene sheet, attached to the porous PDMS frame, contact with each other, and form multiple conductive paths instantly; this results in a rapid increase in the conductivity of the porous graphene-sensitive unit. When the pressure is unloaded, the pores inside the porous graphene-sensitive unit can return to their original shape and the conductive path is disconnected, enabling the porous graphene-sensitive unit have piezoresistive properties.

In the preliminary work of this paper [31], the performance of the sensitive unit had been tested specifically based on its characteristics, such as the response time, pressure detection range, sensitivity, hysteresis and endurance. According to our previous study [31], the response time of the porous graphene-sensitive unit is 40 ms, the force detection range is [0 Pa, 244.4 kPa], the maximum sensitivity is 32.5 kPa^−1^, and the hysteresis is 12.3%. The sensitive unit also displays a high stability over 4000 cycles. In this paper, the experimental results show that the sensor array has a high signal-to-noise ratio and a low detection limit, can quickly perceive the force information, and can sensitively distinguish the small changes of external pressure. The sensor array proposed in this paper can accurately collect the corresponding tactile features for objects, which is also very suitable for hardness and the type detection of different objects.

## 3. Data Acquisition System

### 3.1. Data Acquisition Device

**Figure 5:**
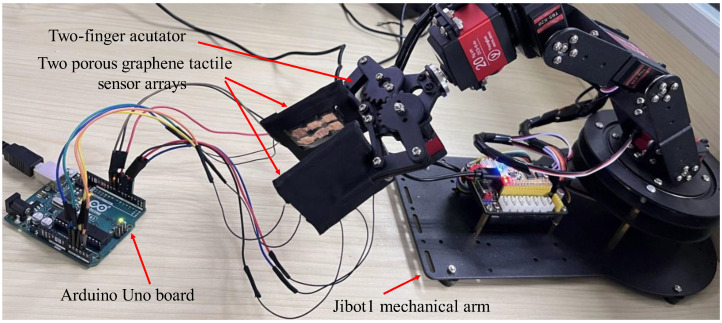
Data acquisition platform.

In this paper, the tactile sensor array, fabricated in Section 2, is installed on the two-finger actuator of the Jibot1 mechanical arm (Jibot 1, Youngbot Co., Hangzhou, China) to construct the data acquisition platform, as shown in Figure 5. Two 2 × 3 sensitive unit arrays, shown in Figure 3, are installed on the left finger and right finger of the mechanical actuator, respectively, and the tactile time sequence feature from the 12 sensitive units of the two sensor arrays are collected in every single data acquisition experiment. In the experiment, the two tactile sensor arrays (shown in Figure 5), connected to the Arduino Uno board (R3, Arduino Co., Italy), are used to detect and perceive the tactile feature information applied to them; in addition, they are used to transmit the output signals from each sensitive unit of the sensor arrays to the computer for storage and processing in real time. In the data acquisition process, the two 2 × 3 sensitive unit arrays are scanned at the same time, and the collected tactile feature information from the 12 sensitive units are stored in the ‘txt’ document in real time by the data acquisition module with a sampling frequency of 25 Hz. The time from the two-finger actuator contacting the object until the signal is received by the data acquisition platform is approximately 0.1 s, which includes two parts. The first part is the response time of each sensitive unit of the flexible tactile sensor array, which is 40 ms; the second part is the time for the Arduino Uno board to collect the signal from the sensitive unit on the two-finger actuator and transmit it to the computer, which is nearly 60 ms. Considering the interference factors that may affect the time from the two-finger actuator touching the object to the signal received by the data acquisition platform, the two-finger actuator is controlled to collect the signal from the tactile sensor array after it reaches the specified position 0.15 s. In the following work, the flexible tactile sensor arrays would be used to realize the hardness-and-type recognition of different objects, based on deep learning algorithms.

### 3.2. Data Acquisition

#### 3.2.1. Objects for Experiments

**Figure 6:**
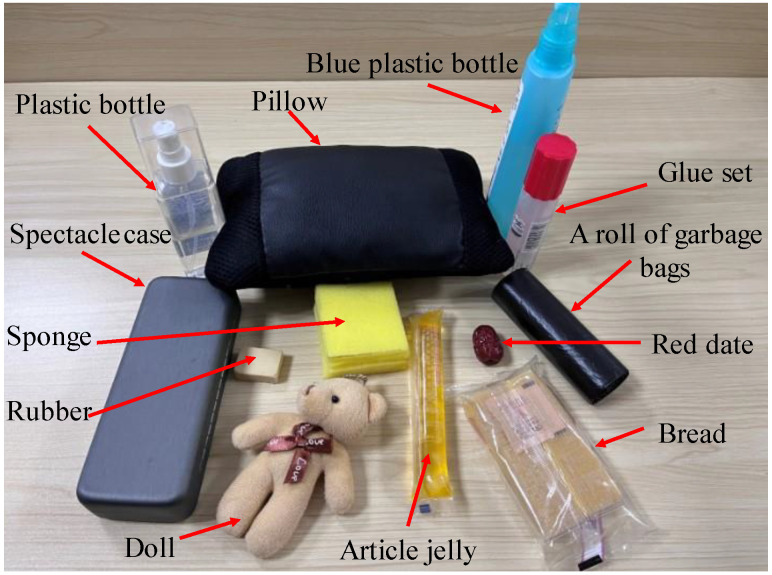
Twelve objects for experiments.

The 12 types of objects, shown in Figure 6, are selected for tactile information perception and recognition experiments. The 12 different objects are a sponge, a pillow, a doll, an article jelly, a piece of bread, a red date, a blue plastic bottle, a glue set, a roll of garbage bags, a rubber, a plastic bottle, and a spectacle case. The hardness and type of the 12 objects would be distinguished by the porous graphene tactile sensor array, based on deep learning algorithms.

To ensure the accuracy of the hardness measurement and realize the effective recognition of the 12 object types, the Shore hardness tester is used to detect and grade the hardness of the 12 different objects. Based on different hardness values, the 12 objects are divided into four categories that are very soft, soft, hard, and very hard. For very soft, its hardness range is [0HA, 20HA]; for soft, its hardness range is [20HA, 40HA]; for hard, its hardness range is [40HA, 60HA]; for very hard, its hardness range is [60HA, 80HA]. The hardness values and the corresponding categories of the 12 objects are shown in Table 1.

#### 3.2.2. Data Acquisition Method

**Figure 7:**
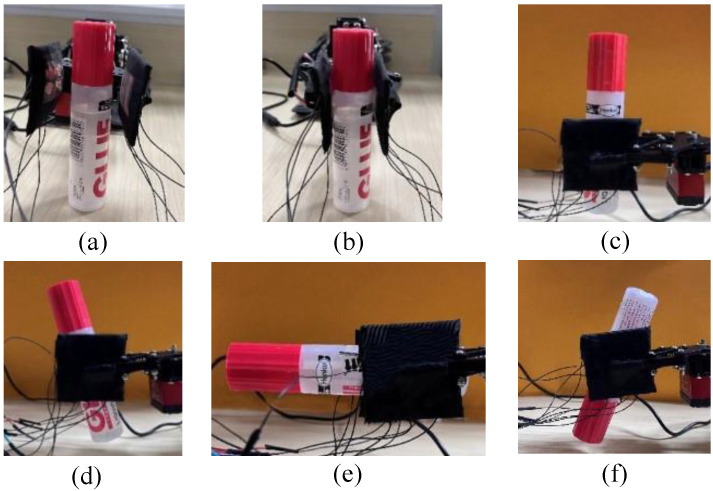
The statuses of gasping an object (glue set) with the two-finger actuator (**a**) Initial state; (**b**) The object is fully touched by the two tactile sensor arrays on the two-finger actuator; (**c**) The object is grasped at the first position; (**d**) The object is grasped at the second position; (**e**) The object is grasped at the third position; (**f**) The object is grasped at the fourth position.

**Figure 8:**
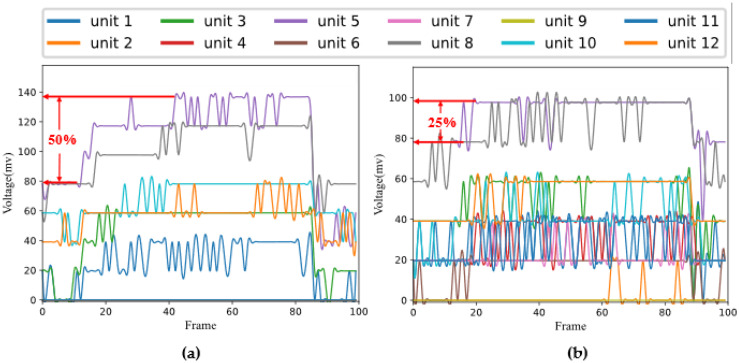
The output voltage time sequence signals of 12 sensitive units from the two sensor arrays for the two objects: (**a**) The voltage time sequence signals collected for the glue set; (**b**) The voltage time sequence signals collected for the sponge.

The porous graphene flexible tactile sensor array, settled on the two-finger actuator of the Jibot1 mechanical arm, is used to build a data acquisition platform and the Arduino Uno board, shown in Figure 5, is used to achieve real-time data acquisition. In the data acquisition process, the two-finger actuator of the mechanical arm should be fully opened, then the object to be detected could be placed between the two fingers and the two-finger actuator should be slowly closed at a constant speed of 3 mm/s to keep both the tactile sensor arrays and the objects stable. To protect the object from being damaged, a data acquisition method, called a small squeezing amount, is proposed in this paper; that is, when the tactile sensor arrays on the two fingers touch the object and generate stable output signals, the two-finger actuator is set to press the object in 2 mm, then it opens slowly at the constant speed of 3 mm/s. The data acquisition experiments should be conducted from four different positions that are chosen randomly for each object, and the experiment for each position is repeated 25 times. Finally, 100 groups of original tactile time sequence data could be collected from each object, which means that each object generates 100 (4 × 25) data samples, and 1200 data samples are obtained from the 12 objects. The statuses of grasping an object (the glue set is given as an example) at four random positions by the two-finger actuator are shown in Figure 7. The output voltage sequence signals from the tactile sensor arrays, collected for both the glue set, whose hardness category is hard, and the sponge, whose hardness category is very soft, are shown in Figure 8, respectively.

When the tactile sensor array touches the object and produces a stable output signal, the two-finger actuator is controlled to slightly squeeze the object. For objects with lower hardness values and a thickness or diameter greater than 2 mm (such as sponge listed in Table 1), the deformation of 2 mm is easily completed. For some very hard objects with low deformability (such as spectacle case listed in Table 1), when the tactile sensor arrays on the two-finger actuator touch the object, the squeezing amount may not reach 2 mm directly. At that time, the torque controller in the two-finger actuator will control the actuator to exert a clamping force positively related to the compression displacement. Due to the interaction of the force, the two tactile sensor arrays mounted on the two-finger actuator would be pressed and reach the amount of 2 mm; then, the electrical signals could be collected. If the thickness or diameter of the soft object is less than 2 mm, under the action of the torque controller, the two-finger actuator would continue to be closed after the object is pressed to the peak point and it could not keep being squeezed. At that time, the two tactile sensor arrays installed on the two-finger actuator would squeeze each other. Meanwhile, the output electrical signal of the tactile sensor array would change slightly at the beginning, then it would be increased significantly. As can be seen from Figure 8, with the increase in the hardness value of the object, the change in the output electrical signal becomes more obvious. The flexible tactile sensor array studied in this paper is mainly applied to common objects in daily life and work, whose thickness or diameter is usually greater than 2 mm. The thickness or diameter of the 12 objects studied in this paper is [10 mm, 60 mm].

The porous graphene sensing unit prepared in this paper is based on the piezoresistive effect. When the pressure is applied on the surface of the sensitive unit, its resistance decreases with the increase in the pressure. At the same time, the corresponding increase in the output voltage could be detected at the end of the Arduino board. Under the same squeezing amount of 2 mm, the higher the hardness of the object, the greater the output voltage of the tactile sensor array, as shown in Figure 8. For example, when the glue set with the hardness of 62 HA is squeezed by 2 mm, the output voltage signal, collected for the glue set from the two tactile sensor arrays with 12 sensitive units increased, approximately 50%, compared with its initial voltage signal (shown in Figure 8a); when the sponge with the hardness of 13 HA is squeezed by 2 mm, the output voltage signal, collected for the sponge from the two tactile sensor arrays with 12 sensitive units, only increased about 25%, compared with its initial voltage signal (shown in Figure 8b). Because the hardness value of the glue set is significantly harder than that of the sponge, the difference in the output voltage signals for the two objects is remarkable.

### 3.3. Data Preprocessing

In the experiment, the two porous graphene tactile sensor arrays are used for tactile data acquisition, and every single tactile sensor array is composed of 2 × 3 sensitive units. To improve the efficiency of data processing and operation, and retain the initial characteristics of the tactile information as much as possible, the tactile time sequence data collected from the two tactile sensor arrays are fused and a sequence data matrix with the dimension of 12 × N, according to time sequence, is constructed, where N is the number of the time frame. Due to the difference between the time dimension N of the collected tactile time sequence data, the Z-score standardization and resampling operation are carried out on the tactile time sequence data to ensure the training effect; the recognition accuracy of the subsequent algorithm models, and all the dimensions of the time sequence data, are unified into 100. After preprocessing, the dimension of the tactile time sequence matrix for the two sensor arrays is 12 × 100. The standardization formula of the Z-score is shown in (1).

(1) $$Z=\frac{X\;-\overset{-}{X}}{\sigma }$$

where, X is the original data, $\overset{-}{X}$ is the mean value of the original data, and σ is the standard deviation. In this paper, the original data X is collected from the porous graphene tactile sensor arrays, and the final dimension of the single tactile time sequence data is 12 × 100.

## 4. Construction of the Residual Network Model

### 4.1. Principle of the Residual Network

The Residual network (ResNet) introduces a residual network structure by adding a shortcut to each residual block so that the gradient flow is directly connected to the bottom layer [35]. When a shallow network has reached saturation accuracy, and the network layer behind it is identity mapping, the network can achieve the best results. While normal networks typically use multiple nonlinear layers to fit the identity map, the ResNet can more easily construct the subsequent network layer as an identity map through a shortcut. This method not only increases the depth of the network, but also preserves the information as much as possible, and reduces the training time and difficulty. The deepening of the network will not lead to the problem of gradient explosion or gradient disappearance; that is, the deeper network will not lead to an increase in training set errors. Therefore, the neural network can extend to a very deep structure and has a relatively strong feature extraction ability.

**Figure 9:**
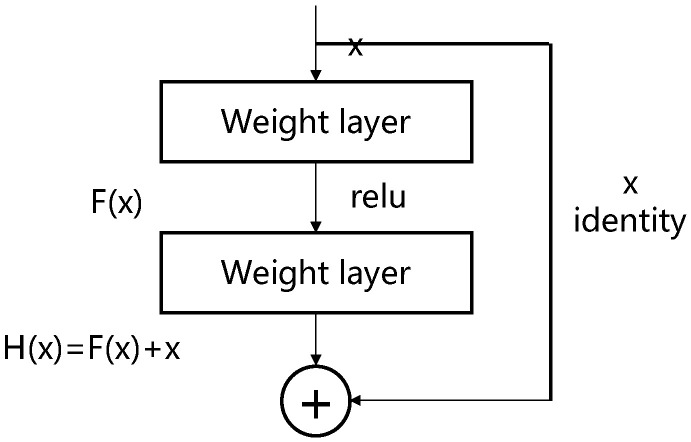
Learning process of the residual block.

The ResNet consists of residual blocks, and its learning process is shown in Figure 9.

The residual block structure can be defined as Equation (2):

(2) $$H\left(x\right)=F\;\left(x,\;\left\{W_{i}\right\}\right)+x$$

where, x and H(x) represent the input and output of the residuals block, respectively; F (x, {W_i_}) is the residuals mapping to be learned. The specific formula is shown in Equation (3):

(3) $$F\;\left(x,\;\left\{W_{i}\right\}\right){=W}_{2}{\;\delta \;(W}_{1}x)$$

where, W_1_ and W_2_ represent the weight matrix of the corresponding layer, and δ represents the simplified ReLU activation function and deviation. F (x, {W_i_}) + x is executed by shortcut join and element addition. Shortcut join is to connect input x directly to the output of the residual block and add elements to the output of the residual block. If the number of channels is changed, and a linear change W_s_ is needed for x when taking a shortcut, then the output H(x) is as follows:

(4) $$H\left(x\right)=F\;\left(x,\;\left\{W_{i}\right\}\right){+W}_{s}x$$

In extreme cases, if the identity mapping is optimal, the network can use an easier way to construct the identity mapping, pushing the residuals F(x) = H(x) − x to zero, which is easier than fitting the identity mapping with multiple non-linear layers. Therefore, the gradient flows directly through these connections, reducing the disappearance or explosion of the gradient, and the training of the deep learning network becomes easier [36,37,38].

Different from the traditional neural network, the ResNet uses the residual structure to enable the output of one layer to directly cross several layers as the input of the next layer, providing a new direction for the problem of overlapping multi-layer networks, so that the error rate of the entire learning model is not reduced, but increases.

In this paper, the tactile time sequence data are used as the input feature vector of the ResNet, a ResNet with excellent adaptability and generalization ability is constructed, and the hardness-and-type recognition for the 12 objects would be realized with high precision, based on the porous graphene flexible tactile sensor arrays.

### 4.2. The ResNet Model for the Tactile Sensor Array

The intelligent actuator can perceive the typical features of objects by setting the flexible tactile sensor on itself, which can help the intelligent robot to accurately complete object recognition and human–computer interaction. To improve the recognition ability of the tactile feature information detected by the porous graphene flexible tactile sensor array, a ResNet model is constructed based on the deep learning algorithm theory; the tactile time sequence feature from the 12 objects with different hardness values, collected by the tactile sensor arrays, would be precisely recognized and classified into four categories (very soft, soft, hard, very hard) by the ResNet. Meanwhile, the 12 object types would be accurately identified by the same ResNet model. 

**Figure 10:**
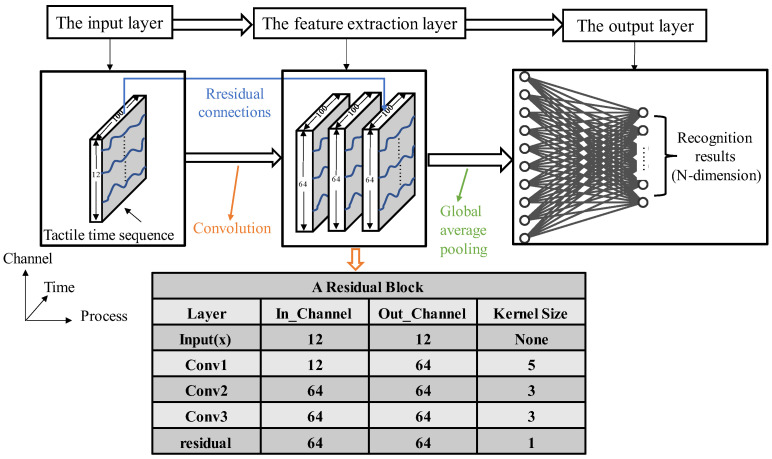
The residual network model for the tactile sensor.

Based on the tactile time sequence data collected by the porous graphene flexible tactile sensor, the residual network with an input layer, a feature extraction layer, a global average pooling layer, and an output layer, is constructed in this paper. Its structure is shown in Figure 10. The input data of the input layer is the preprocessed tactile time sequence from different objects, which means that the dimension of the input layer, based on a single data sample, is (1, 12, 100), where, “1” represents a single sample, “12” represents the number of channels, and “100” is the length of the time sequence. The feature extraction layer includes a residual block that is composed of three convolution kernels whose sizes are 5, 3, and 3, respectively. The convolution kernel with the size of 1 is used to expand the number of channels, to keep the same number of data channels for the residual operation. The output of the feature extraction layer is taken as the input of the global average pooling layer, and the output of the pooling layer is the feature maps of multiple channels that correspond to different categories, respectively. The softmax function is used to map the feature maps of the channels to the exact probability values of different categories, and the probability values are the output of the whole ResNet.

## 5. Analysis and Discussion of Experimental Results 

### 5.1. Hardness Recognition of Objects Based on the ResNet Model

Experiments that use the porous graphene flexible tactile sensor array to grasp the 12 objects (listed in Figure 6 and Table 1) under the small squeezing amount of 2 mm are conducted, and 1200 groups of original tactile data are obtained; the original tactile data are standardized and resampled by the Z-score method introduced in Section 3.3. In the 1200 groups of samples, there are 300 groups of very soft objects, soft objects, hard objects, and very hard objects, respectively. The 1200 samples are randomly divided into a training set and testing set at a ratio of 7:3. In both the training set and the testing set, the number of samples from the four hardness categories (very soft, soft, hard, and very hard) are the same. On this basis, the ResNet model is built by PyTorch, which is the deep learning library. In the training procedure of the ResNet, the CrossEntropyLoss function is selected as the loss function, the Adam optimizer is used to optimize the model, and the gradient is calculated by information back propagation, so as to update the parameters of the ResNet model. The initial learning rate of the ResNet is 0.0001, the batch input size is 16, and the training iterations is 100. After training, the testing samples are used to verify the performance of the ResNet model. Finally, the average recognition accuracy of 4 hardness categories (very soft, soft, hard, very hard) from the 12 objects is obtained; its value is 100%. The results indicate that the ResNet model, constructed in this paper, has excellent feature extraction capabilities and can be very well applied to the flexible tactile sensor to recognize the hardness of different objects. The calculation formula of accuracy is shown in (5) [39]:

(5) $$\mathrm{accuracy}=\frac{1}{N}\sum_{i=1}^{N}I\left(y^{\left(i\right)}={\hat{y}}^{\left(i\right)}\right)$$

where, N denotes the number of testing samples, $y^{\left(i\right)}$ denotes the true category of the i-th sample, ${\hat{y}}^{\left(i\right)}$ denotes the output category of the ResNet model for the i-th sample; function I( ) is used to estimate whether $y^{\left(i\right)}$ and ${\hat{y}}^{\left(i\right)}$ are equal; if they are equal, its value is 1, otherwise its value is 0.

In order to further verify the ability of the ResNet model to recognize the tactile time sequence data for the porous graphene flexible sensor from different objects, the MLP, LeNet, MCDCNN, and ENCODER network models are built based on the same dataset, respectively. These four models are also used to recognize the hardness for different objects. The main parameters of the models include the number of layers (Layers), the number of convolution layers (Conv), the feature mapping layer (Feature), the pooling layer (Pooling) and the activation layer (Activate); their specific values are shown in Table 2.

**Figure 11:**
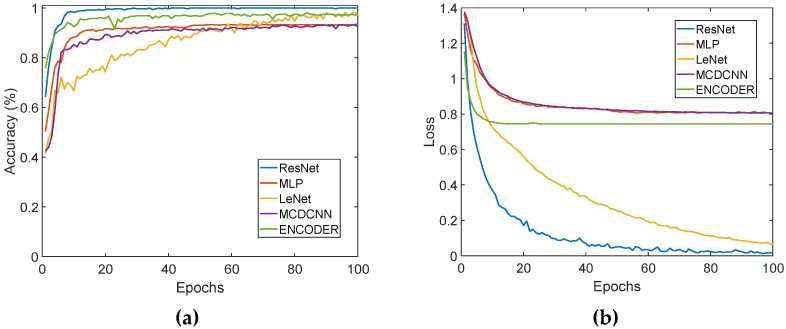
Learning curves of the five models based on four category samples: (**a**) The average accuracies of 4 hardness categories based on the five models; (**b**) Loss values of the five models.

In the training process, the iteration times of the five network models, listed in Table 2, are all 100, and their learning curves are shown in Figure 11. It can be seen from Figure 11a that the average recognition accuracy of the four-hardness categories, based on the ResNet model, reaches 100% at its best; the average recognition accuracies, based on the MLP, LeNet, MCDCNN, and ENCODER, are 93.6%, 98.3%, 93.3%, and 98.1%, respectively. All of the above results show that the porous graphene flexible tactile sensor, proposed in this paper, has a very good performance and can accurately perceive the tactile feature loaded on its surface; additionally, it can prompt the five models and obtain a high recognition accuracy for the four-hardness categories. The results also show that the ResNet model is much more suitable for hardness recognition than the other four models. In Figure 11b, the loss values of the ResNet, MLP, LeNet, MCDCNN, and ENCODER are 0.012, 0.806, 0.066, 0.806, and 0.744, respectively, which also indicate that the ResNet model has better performance than the others in the recognition of tactile feature. That is mainly because the ResNet has a more flexible structure, which can obtain a more tactile time sequence feature. In addition, different from other models, the ResNet adopts a global average pooling layer, which can preserve the tactile feature information extracted by the previous convolution layers, then achieve a higher recognition accuracy.

**Figure 12:**
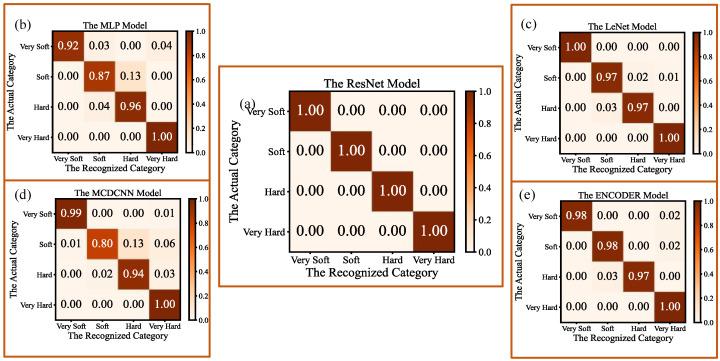
Confusion matrixes of recognition results for the four-hardness categories based on the five models: (**a**) The confusion matrix for the ResNet model; (**b**) The confusion matrix for the MLP model; (**c**) The confusion matrix for the LeNet model; (**d**) The confusion matrix for the MCDCNN model; (**e**) The confusion matrix for the ENCODER model.

The confusion matrixes of the recognition results for the four hardness categories (very soft, soft, hard, very hard), based on the five models, are shown in Figure 12. As can be seen from Figure 12, all of the five models obtain higher recognition accuracies for objects with very soft and very hard categories, and gain lower recognition accuracies for the other hardness categories. For example, the recognition accuracies for very soft and very hard objects, based on the MCDCNN algorithm model, are 99% and 100%, respectively, and those for soft and hard objects are 80% and 94%, respectively; the recognition accuracies for very soft and very hard objects, based on the LeNet model algorithm, are both 100%, and those for soft and hard objects are both 97%. This is mainly due to the fact that, during the small squeezing process of 2 mm, the hardness level of each very soft object or each very hard object has a larger span, and more obvious signal frequency features could be easily obtained by the tactile sensor array. Therefore, the differences between the collected tactile feature information from the very soft and the very hard objects are much more significant than those from the soft and the hard objects, and they are more easily distinguished.

All the experimental results indicate that the flexible tactile sensor, fabricated in this paper, has an excellent perception capability and can accurately perceive the tactile feature information from different objects with different hardness levels; in addition, the ResNet model, with great classification capabilities, can very precisely recognize the hardness categories of different objects based on tactile time sequence feature information, detected by the tactile sensor arrays.

### 5.2. Type Recognition of Objects Based on the Five Models

**Figure 13:**
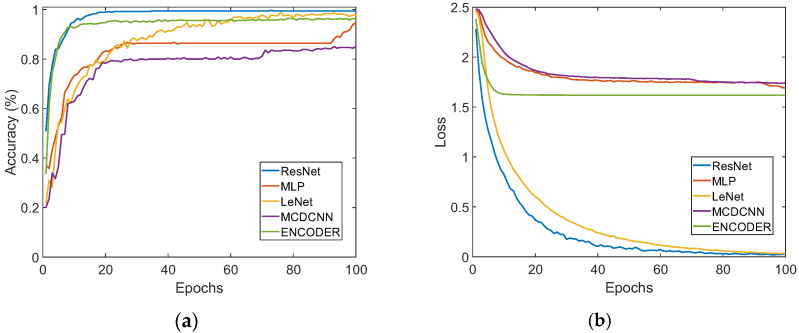
Learning curves of the five models based on the 12 object types. (**a**) Learning curves of the five models. (**b**) Loss values of the five models.

The flexible tactile sensor can also recognize different types of objects based on machine learning algorithm models, according to the surface characteristics of different objects. In order to verify the effectiveness of the five algorithm models used for the type recognition of objects, based on the same time sequence signal detected by the tactile sensor arrays in Section 5.1, the 1200 samples collected from the 12 object types (shown in Figure 6) are divided into 12 subsets; each subset includes 100 samples from one object. This means that there are 12 types in the 1200 samples. The 1200 samples are randomly divided into a training set and testing set, at a ratio of 7:3. The training set consists of 840 samples from the 12 object types (i.e., 70 samples for each object), and the testing set consists of 360 samples from the 12 object types (i.e., 30 samples per object). Based on the samples containing feature information from the 12 object types, the experiments for the classification and recognition of the 12 object types are carried out by the same five network models (ResNet, MLP, LeNet, MCDCNN, and ENCODER) built in Section 5.1, respectively. The learning curves of the five network models for recognizing the 12 object types are shown in Figure 13, respectively. 

**Figure 14:**
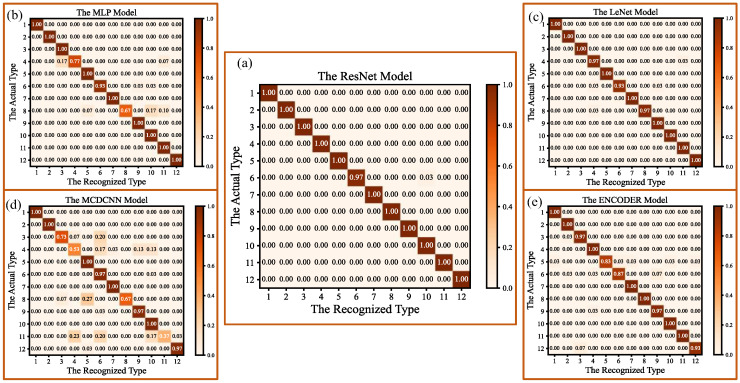
Confusion matrix of recognition results for the 12 object types, based on the five models.

In Figure 13a, the average recognition accuracies for the 12 object types, based on the ResNet, MLP, LeNet, MCDCNN, and ENCODER models, are 99.7%, 94.7%, 98.9%, 85.0% and 96.4%, respectively. This implies that the recognition accuracy of the 12 object types, based on the ResNet model, is still higher than that of other algorithm models. As shown in Figure 13b, the loss values of the five models are 0.019, 1.691, 0.032, 1.739 and 1.619, and the loss value of the ResNet is still the lowest. The confusion matrixes for the classification and recognition results for the 12 object types, based on the five algorithm models, are shown in Figure 14. The label numbers (1–12) correspond to the 12 object types in the confusion matrixes, are listed in Table 3. As shown in Figure 14, the recognition accuracies for sponge, pillow and rubber, based on the five models, are all up to 100%. This is mainly because the hardness values of sponge and pillow belong to the categories of very soft, and the hardness value of rubber belongs to the category of very hard. This means that the hardness values of the three object types are obviously different from other objects, so it is easy to perceive their obvious features from each other. It is also concluded that the flexible tactile sensor can availably detect the feature information from different object types, and the ResNet model with a great performance can efficiently capture different signal frequency features and realize the high-precision recognition of multi-type objects.

## 6. Conclusions

A novel porous graphene flexible tactile sensor array, used for hardness-and-type recognition, is proposed and fabricated in this paper. Based on the tactile sequence feature, detected by the tactile sensor arrays for different objects, five deep learning models, ResNet, MLP, LeNet, MCDCNN, and ENCODER, are constructed to conduct the recognition of the 4 hardness categories and 12 object types, respectively. The average recognition accuracy of the 4 hardness categories, based on the ResNet model, is 100%, which is 6.4 points, 1.7 points, 6.7 points, and 1.9 points higher than those of the other four models, respectively. Meanwhile, the average recognition accuracy of the 12 object types, based on the ResNet model, is 99.7%, which is 5 points, 0.8 points, 14.7 points, and 3.3 points higher than those of the other four models, respectively. The experimental results denote that the porous graphene flexible tactile sensor array, proposed in this paper, is excellently capable of detecting the tactile time sequence feature; in addition, the ResNet algorithm model, with a great generalization capability and adaptivity can be very well applied to the hardness-and-type recognition of different objects, based on the tactile time sequence information collected by the flexible tactile sensor array.

## Figures and Tables

**Table 1:** Hardness values and categories of the 12 objects.

Object	Hardness Value	Hardness Category	Object	Hardness Value	Hardness Category
Sponge	13HA	Very soft	Blue plastic bottle	67HA	Hard
Pillow	15HA	Very soft	Glue set	62HA	Hard
Doll	11HA	Very soft	A roll of garbage bags	58HA	Hard
Article jelly	32HA	Soft	Rubber	80HA	Very hard
Bread	34HA	Soft	Plastic bottle	78HA	Very hard
Red date	27HA	Soft	Spectacle case	82HA	Very hard

**Table 2:** Parameters of the five models.

Model	Layers	Conv	Feature	Pooling	Activate
MLP	4	0	FC	None	ReLU
LeNet	4	2	FC	Max	ReLU
MCDCNN	4	2	FC	Max	ReLU
ENCODER	5	3	Att	Max	PReLU
ResNet	4	3	GAP	Avg	ReLU

**Table 3:** The label numbers of the 12 object types.

Object	Label Number	Object	Label Number	Object	Label Number
Sponge	1	Bread	5	A roll of garbage bags	9
Pillow	2	Red date	6	Rubber	10
Doll	3	Blue plastic bottle	7	Plastic bottle	11
Article jelly	4	Glue set	8	Spectacle case	12

## Data Availability

The data used to support the study are available upon request to the corresponding author.
